# Clinical and body composition parameters as predictors of response to chemotherapy plus PD-1 inhibitor in gastric cancer

**DOI:** 10.3389/fimmu.2025.1685592

**Published:** 2025-10-07

**Authors:** Chenfei Zhou, Yan Sun, Tao Liu, David P. J. van Dijk, Wenqi Xi, Jinling Jiang, Liting Guo, Feng Qi, Xuekun Zhang, Mengfan Jia, Jun Ji, Zhenggang Zhu, Sander S. Rensen, Steven W. M. Olde Damink, Jun Zhang

**Affiliations:** ^1^ Department of Oncology, Ruijin Hospital, Shanghai Jiao Tong University School of Medicine, Shanghai, China; ^2^ Department of Surgery, Institute of Nutrition and Translational research in Metabolism (NUTRIM), Maastricht University, Maastricht, Netherlands; ^3^ Department of Gastrointestinal Surgery, The First Affiliated Hospital of Zhengzhou University, Zhengzhou, China; ^4^ Department of Radiology, Ruijin Hospital, Shanghai Jiao Tong University School of Medicine, Shanghai, China; ^5^ Shanghai Institute of Digestive Surgery, Ruijin Hospital, Shanghai Jiao Tong University School of Medicine, Shanghai, China; ^6^ Department of Gastrointestinal Surgery, Ruijin Hospital, Shanghai Jiao Tong University School of Medicine, Shanghai, China; ^7^ Department of General, Visceral, and Transplantation Surgery, University Hospital Essen, Essen, Germany; ^8^ Shanghai Key Laboratory of Gastric Neoplasms, Shanghai, China

**Keywords:** gastric cancer, immune checkpoint inhibitors, clinical prediction model, tumor regression grade, body composition

## Abstract

**Background:**

Predicting the treatment efficacy of programmed cell death protein 1 (PD-1) inhibitors is crucial for guiding optimal treatment plans and preventing unnecessary complications for cancer patients. We aimed to develop a prediction model using clinical and body composition parameters to identify gastric cancer (GC) patients who would respond to chemotherapy plus PD-1 antibody.

**Methods:**

Clinical data of GC patients treated with chemotherapy plus PD-1 antibody (immunotherapy cohort, n = 120) or chemotherapy alone (chemotherapy cohort, n = 82) following surgical resection were reviewed as the training set. Patients treated with chemotherapy plus PD-1 antibody at an external center were included as the validation set (n = 43). Tumor regression grade (TRG) was recorded and classified as TRG0/1 or TRG2/3 during analysis. Body composition parameters were assessed on computed tomography images at the third lumbar vertebral level using the SliceOmatic software. Univariate and multivariate analyses were performed to identify parameters associated with TRG0/1, and then a logistic regression model was developed to stratify patients into the good and poor response groups.

**Results:**

In the training set, clinical and body composition parameters between the immunotherapy cohort and chemotherapy cohort were similar. Skeletal muscle radiation attenuation (SMRA), neutrophil-to-lymphocyte ratio (NLR), and weight loss were associated with TRG0/1 in the immunotherapy cohort. Subcutaneous adipose tissue index (SATI) and metastasis were identified in the chemotherapy cohort. A logistic regression model was developed to stratify immunotherapy cohort patients into two response groups with an area under the receiver operating characteristic curve (AUC) value of 0.728. In the immunotherapy cohort, patients stratified as good responders showed a higher TRG0/1 rate (37/55, 67.3%) than poor response patients (18/65, 27.7%, *p* < 0.001) and had better overall survival (*p* = 0.001). In the external validation set, patients stratified using the clinical model as good responders also showed a higher TRG0/1 rate (14/18, 77.8%) than poor response patients (9/25, 36.0%, *p* = 0.012).

**Conclusion:**

The prediction model consisting of SMRA, NLR, and weight loss could help identify GC patients who respond well to chemotherapy plus PD-1 antibody.

## Background

Gastric cancer (GC) is among the most malignant diseases worldwide, with over 40% of new cases occurring in China (1). Moreover, approximately 80% of Chinese GC patients are diagnosed at an advanced stage (2). Median overall survival for GC patients with unresectable locally advanced disease or distant metastasis is barely over 12 months, and the 5-year overall survival rate is below 40% (3).

Novel treatment strategies and drugs are now under investigation to meet the urgent needs of GC patients. Programmed cell death protein 1 (PD-1) antibodies have shown efficacy in various cancers and have become a key treatment in some cases (4). For advanced gastric cancer (AGC) patients, the ATTRACTION-2 trial showed the antitumor efficacy of nivolumab monotherapy in late-stage patients as salvage treatment (5). Nowadays, multiple randomized phase 3 trials have demonstrated that combining PD-1 antibody with chemotherapy can improve the survival of HER2-negative AGC patients as a first-line regimen compared to chemotherapy alone (6–8).

Predictive biomarkers are important for guiding optimal treatment plans for cancer patients by identifying those who would respond to specific therapeutics. For AGC patients, PD-1 antibody plus chemotherapy significantly improved overall survival versus chemotherapy alone in patients with PD-L1 combined positive score (CPS) ≥5 (9). Microsatellite instability high (MSI-H) is another pan-cancer predictive biomarker for PD-1 antibodies. However, there are some limitations of these biomarkers. The prevalence of PD-L1 CPS ≥5 is approximately 10%–30% in GC (10, 11), and its expression is detected by immunohistochemistry, which can be affected by the type of antibody, the staining procedure, and the assessment of pathologists (12). The prevalence of MSI-H is also relatively low in GC, and not all AGC patients with MSI-H can achieve an objective response to PD-1 antibodies (13). For most patients who do not have these biomarkers, whether they can benefit from the therapy is still under investigation. However, the risk of immune-related adverse events (irAEs) should be noted (14). Therefore, more novel strategies are urgently needed to guide immunotherapy of GC.

The association between body composition and clinical outcomes of cancer patients has been thoroughly investigated (15–17). Based on images of computed tomography (CT), a routine examination for cancer patients, body composition parameters can be objectively analyzed without significantly increasing costs (18). Aberrant changes of body composition parameters, such as low skeletal muscle radiation attenuation (SMRA; i.e., myosteatosis) and low skeletal muscle mass (i.e., sarcopenia), have been recognized as long-lasting results of tumor and host interaction (19). Tumor cells change the metabolism of host tissues and modulate immune cell activation (20). Conversely, skeletal muscle and adipose tissues with aberrant metabolic conditions can affect the host immune system (21, 22). Therefore, body composition can represent the homeostasis of the host immune system and consequently influence response to PD-1 antibody-based therapy. Associations between body composition and outcomes of PD-1 antibodies have been reported in melanoma and lung cancer patients (23), but it has yet to be integrated into a clinically applicable prediction model for GC.

Tumor regression grade (TRG) is an objective outcome of systemic treatment that is closely associated with patients’ survival (17). This study assessed the association of body composition and clinical factors with the pathological response in GC patients treated with chemotherapy plus PD-1 antibody, aiming to develop a multivariate prediction model to identify GC patients who would benefit from this combination therapy.

## Methods

### Study population

**Figure 1:**
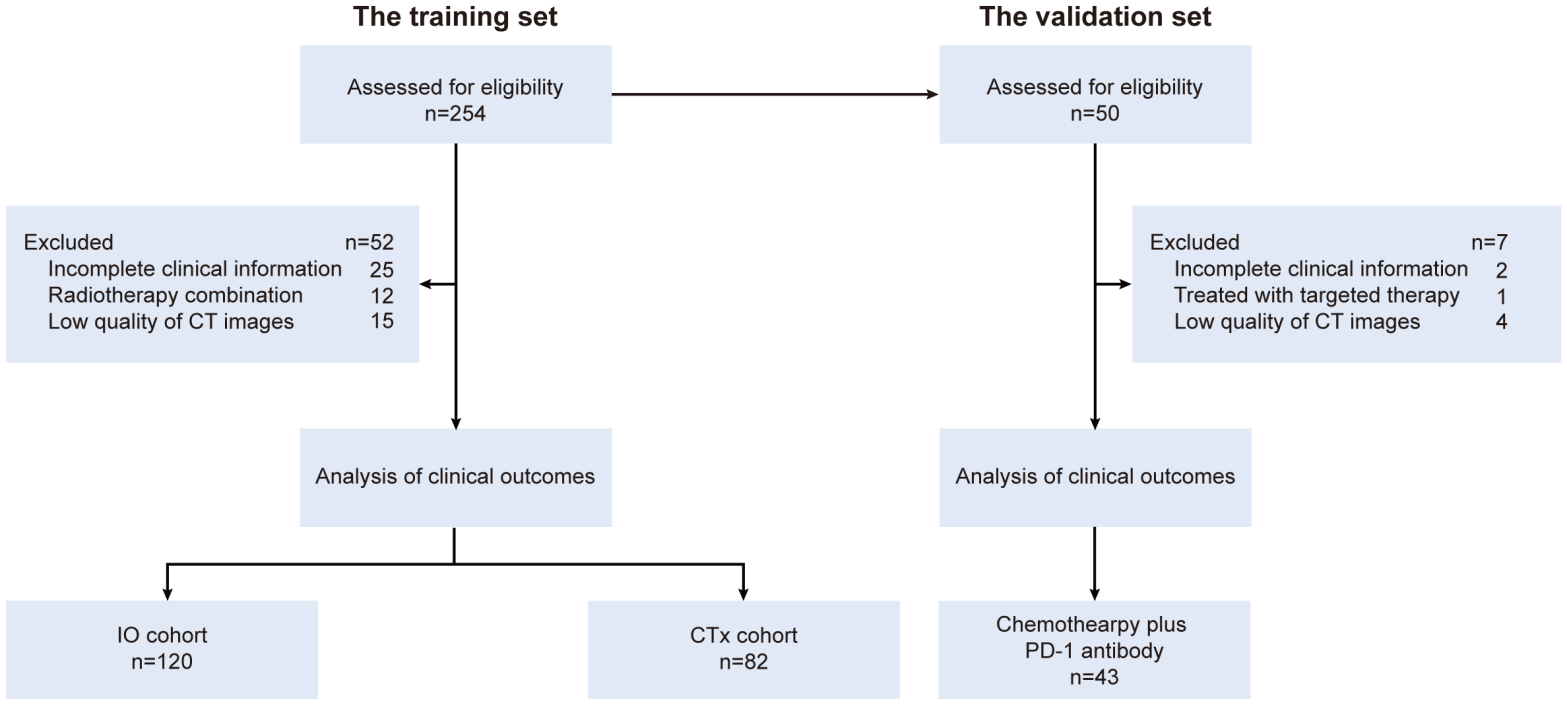
Study profile of patients’ enrollment and analysis.

GC patients treated at the Department of Oncology, Ruijin Hospital, from January 2017 to December 2022 were reviewed as the training set (n = 254). Patients who received chemotherapy plus PD-1 antibody were assigned to the “IO cohort”, and those who received chemotherapy alone during the same period were assigned to the “CTx cohort” as the reference. An external validation set consisted of GC patients (n = 50) who underwent chemotherapy plus PD-1 antibody following surgical resection at the Department of Gastrointestinal Surgery, the First Affiliated Hospital, of Zhengzhou University, from April 2021 to January 2024 were included (**Figure 1**). The major enrollment criteria were as follows: pathologically confirmed gastric adenocarcinoma, potentially resectable locally advanced disease with or without distant metastasis, radical resection with D2 lymphadenectomy or palliative gastrectomy performed after systemic treatment, and availability of images of enhanced computed tomography scans at the third lumbar vertebral (L3) level before treatment (within 1 month before initiation of systemic treatment). The exclusion criteria were incomplete clinical information, having been treated with radiation therapy before surgery, and poor quality of CT scan images. This study was approved by the Ruijin Hospital Ethics Committee (2023, No. 132). A waiver of consent form was obtained.

### Treatment procedures

Fluoropyrimidine-based chemotherapy regimens were administered. Triplet regimens included FLOT (docetaxel and oxaliplatin plus 5-fluorouracil), POS (paclitaxel and oxaliplatin plus S-1), and POX (paclitaxel and oxaliplatin plus capecitabine). Doublet regimens included SOX (oxaliplatin plus S-1) and XELOX (oxaliplatin plus capecitabine). A standard dosage of each cytotoxic drug was administered at the first cycle, and dose reduction was performed following clinical protocol if necessary. PD-1 antibodies were administered following standard dose and interval, including pembrolizumab, nivolumab, camrelizumab, and sintilimab. Chemotherapy regimens with a 2-week interval comprised four cycles, and regimens with a 3-week interval comprised three cycles before surgery. Surgery was performed 3–4 weeks after systemic treatment. Treatment cycles were extended if it was difficult to perform the surgical resection of the primary lesion of the stomach based on the assessment of the surgeons. Patients were re-evaluated using an enhanced CT scan every 8 to 9 weeks.

### Assessment of treatment efficacy

Pathological response after surgery was recorded as TRG and assessed by pathologists who were blinded to the study. The consensus criteria recommended by the Chinese Society of Clinical Oncology gastric cancer guideline were used: TRG0, the absence of visible cancer cells, including lymph nodes (complete response); TRG1, the presence of single cell or few small clusters of cancer cells (near-complete response); TRG2, the presence of residual cancer cells with evident tumor regression but a larger number of single cells or groups of cancer cells (partial response); and TRG3, the presence of extensive residual cancer without evident tumor regression (poor or no response) (24). Tumor regression grades were classified as TRG0/1 or TRG2/3 during the following analysis. The overall survival (OS) of patients was monitored. OS was defined as the time from diagnosis to death. The follow-up period was defined as the time from the initial diagnosis until the occurrence of death, loss to follow-up, or the end of the study period (June 30, 2025), whichever occurred first.

### Body composition analysis

A single baseline transverse CT scan image at the middle L3 level of each patient for body composition analysis was collected from the picture archiving and communication system. Skeletal muscle and adipose tissue were segmented by the SliceOmatic software (v5.0, TomoVision) using predefined Hounsfield unit (HU) ranges for skeletal muscle (SM; −29 to 150 HU), visceral adipose tissue (VAT; −150 to −50 HU), and subcutaneous adipose tissue (SAT; −190 to −30 HU). Mean radiation attenuation (RA) values of skeletal muscle (SMRA), visceral adipose tissue (VATRA), and subcutaneous adipose tissue (SATRA) were calculated. The cross-sectional areas of SM, VAT, and SAT were normalized to the patient’s height to calculate indices (cm^2^/m^2^) for SM (SMI), VAT (VATI), and SAT (SATI).

### Other clinical parameters

Clinical data, including age, gender, height, weight, and clinical TNM (cTNM) stage, were recorded. Body weight loss within 6 months before diagnosis was recorded based on medical history taking. A cut-off of 5% body weight loss within 6 months before diagnosis was used to stratify patients into high or low weight loss. Laboratory results were recorded before treatment, including neutrophil count, lymphocyte count, and prealbumin levels. The neutrophil-to-lymphocyte ratio (NLR) was calculated.

### Statistical analysis

Continuous data are described as median values with range. Differences in patients’ characteristics were analyzed using the Mann–Whitney U test and χ^2^ test, where appropriate. Univariate binary logistic regression analysis was performed to identify parameters associated with TRG. Bivariate correlation (Pearson’s) was performed to analyze associations among clinical parameters. Parameters that showed significance (*p* < 0.05) in univariate analyses were selected as the candidate variables and entered into multivariate regression models to establish a logistic regression model to stratify patients into the good and poor response groups. Cut-off values of parameters and the prediction model were determined using receiver operating characteristic (ROC) curves. The cut-off for the prediction model was determined using Youden’s index, which is defined as (sensitivity + specificity − 1). The value corresponding to the maximum Youden’s index was selected as the optimal cut-off. The association between the prediction model and OS was evaluated using the Kaplan–Meier analysis. A *p*-value less than 0.05 was considered significant. Data analyses were performed using the SPSS 22.0 software (Chicago, IL, USA).

## Results

### Clinical characteristics of patients

A total of 202 eligible patients constituted the training set, including 120 patients treated with chemotherapy plus PD-1 antibody (IO cohort) and 82 patients treated with chemotherapy alone (CTx cohort). Among them, 131 (64.9%) were male, and the median age was 62.0 years. Baseline clinical characteristics were generally similar between the IO cohort and CTx cohort, including gender-specific height, weight, and body mass index (BMI). The median time interval from perioperative treatment initiation to surgery was 3.3 months (1.8 to 15.0 months), and the median number of treatment cycles was 3 in both cohorts. The median follow-up time was 37.0 months (3.9 to 101.9 months). Five-year OS rates were 66.0% in the IO cohort and 52.5% in the CTx cohort. In the validation set, 37 patients (86.0%) were male, with a median age of 63.0 years, showing similar characteristics to the IO cohort of the training set (**Table 1**).

**Table 1:** Clinical characteristics of gastric cancer patients in the training set and validation set.

Clinical characteristics	Training set			Validation set (n = 43)
	All patients	IO cohort (n = 120)	CTx cohort (n = 82)	
Age (years)	62.0 (25.0, 81.0)	63.0 (29.0, 78.0)	61.0 (25.0, 81.0)	63.0 (48.0, 78.0)
Gender Male/female (%)	131/71 (64.9/35.1)	84/36 (70.0/30.0)	47/35 (57.3/42.7)	37/6 (86.0/14.0)
Height (cm)	168.0 (145.0, 185.0)	168.0 (145.0, 183.0)	167.0 (150.0, 185.0)	169.0 (148.0, 187.0)
Male	170.0 (155.0, 185.0)	170.0 (155.0, 183.0)	170.0 (159.0, 185.0)	170.0 (150.0, 187.0)
Female	160.0 (145.0, 173.0)	160.0 (145.0, 168.0)	160.0 (150.0, 173.0)	160.0 (148.0, 165.0)
Weight (kg)	63.0 (42.1, 99.0)	63.5 (42.1, 94.0)	61.5 (45.0, 99.0)	65.0 (50.0, 110.0)
Male	65.0 (45.0, 99.0)	65.0 (48.0, 94.0)	66.0 (45.0, 99.0)	65.0 (40.0, 110.0)
Female	55.0 (42.1, 79.0)	54.3 (42.1, 76.9)	55.0 (46.0, 79.0)	58.0 (50.0, 65.0)
BMI (kg/m^2^)	22.21 (13.44, 34.26)	22.33 (17.01, 31.98)	21.96 (13.44, 34.26)	22.65 (18.36, 35.91)
Male	22.62 (13.44, 34.26)	22.78 (17.01, 31.98)	22.13 (13.44, 34.26)	22.60 (18.36, 35.91)
Female	21.34 (17.52, 32.05)	21.31 (17.52, 29.76)	21.50 (18.07, 32.05)	23.26 (19.53, 25.79)
Weight loss (%)	3.07 (0, 20.62)	3.25 (0.0, 20.62)	2.61 (0.0, 16.36)	0.0 (0.0, 13.9)
cTNM, n (%)				
II	0 (0.0)	0 (0.0)	0 (0.0)	7 (16.3)
III	103 (51.0)	50 (41.7)	53 (64.6)	32 (74.4)
IV	99 (49.0)	70 (58.3)	29 (35.4)	4 (9.3)
Chemotherapy regimens, n (%)				
Triplet	81 (40.1)	45 (37.5)	36 (43.9)	0 (0.0)
Doublet	121 (59.9)	75 (62.5)	46 (56.1)	43 (100)
Treatment cycles	3 (2, 22)	3 (2, 22)	3 (3, 8)	3 (2, 7)
Tumor regression grade (TRG), n (%)				
TRG 0	20 (9.9)	15 (12.5)	5 (6.1)	11 (25.6)
TRG 1	62 (30.7)	40 (33.3)	22 (26.8)	12 (27.9)
TRG 2	92 (45.5)	51 (42.5)	41 (50.0)	11 (25.6)
TRG 3	28 (13.9)	14 (11.7)	14 (17.1)	9 (20.9)

BMI, body mass index; IO cohort, immunotherapy cohort; CTx, cohort, chemotherapy cohort; cTNM, clinical TNM.

### SMRA, NLR, and weight loss are associated with TRG0/1 in the IO cohort

**Figure 2:**
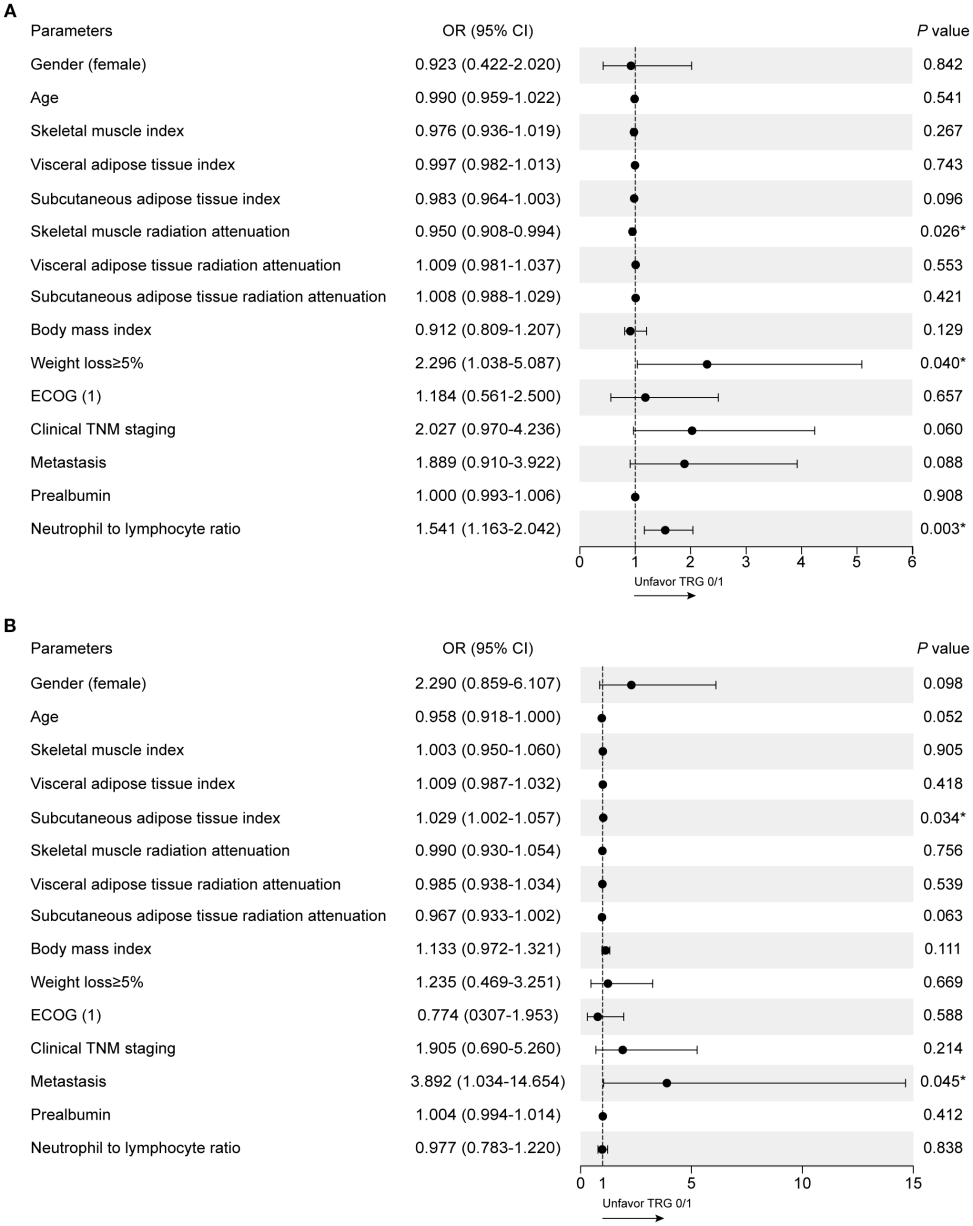
Clinical factors associated with TRG0/1 using univariate logistic regression models. **(A)** Univariate analysis in the IO cohort. **(B)** Univariate analysis in the CTx cohort.

Univariate binary logistic regression analysis was performed to identify parameters associated with TRG0/1 in the training set (**Supplementary Table S1**). For the IO cohort, SMRA (OR = 0.950, 95%CI 0.908–0.994, *p* = 0.026), weight loss ≥5% (OR = 2.296, 95%CI 1.038–5.087, *p* = 0.040), and NLR (OR = 1.541, 95%CI 1.163–2.042, *p* = 0.003) were significantly associated with TRG0/1 (**Figure 2A**). For the CTx cohort, SATI and metastasis were significantly associated, but the three parameters identified in the IO cohort were not (**Figure 2B**). There were no significant differences in values of body composition parameters and laboratory results between the IO cohort and the CTx cohort (**Supplementary Table S2**).

### Establishment of the prediction model

To establish the prediction model associated with the pathological response of chemotherapy plus PD-1 antibody, SMRA, weight loss ≥5%, and NLR were selected and further analyzed using multivariate logistic regression analysis. All three parameters were entered into the equation (**Supplementary Table S1**), and no significant correlations among SMRA, NLR, and weight loss were detected (**Supplementary Table S3**). Then, a logistic regression model was developed: Logit(*p*) = 1.407 − 0.055 × SMRA + 0.397 × NLR + 0.749 × weight loss (<5% = 0, ≥5% = 1). By ROC analysis, the area under the receiver operating characteristic curve (AUC) of the clinical model to predict TRG0/1 was 0.728 (*p* < 0.001) and was higher than that of SMRA (AUC = 0.644), NLR (AUC = 0.678), and weight loss ≥5% (AUC = 0.590) as a single parameter (**Supplementary Figure S1**).

### The efficacy of the prediction model in the training set

**Figure 3:**
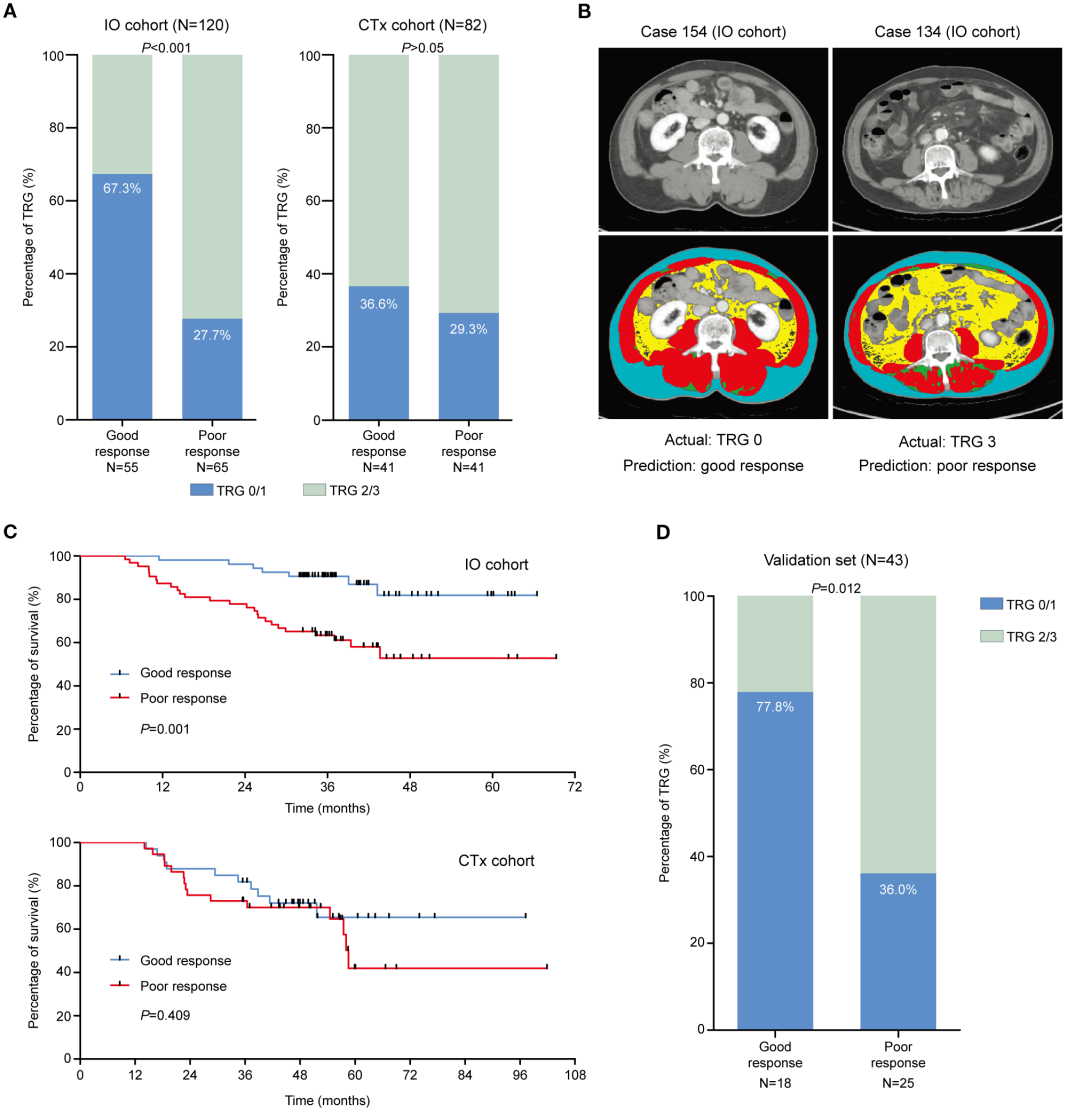
The efficacy of the prediction model in the training set. **(A)** TRG0/1 rates of patients who were stratified as good and poor responders in the IO cohort and CTx cohort, respectively. **(B)** The representative images of patients who were stratified using the prediction model in the IO cohort. **(C)** The survival of patients who were stratified as good and poor responders in the IO cohort and CTx cohort, respectively. **(D)** TRG0/1 rates of patients who were stratified as good and poor responders in the validation set.

The cut-off of the prediction model was determined as 0.095, which could stratify patients into the good response group and the poor response group. For the IO cohort, 55 patients were stratified into the good response group, and 37 of them achieved TRG0/1 (37/55, 67.3%), which was significantly higher than patients who were stratified into the poor response group (18/65, 27.7%, *p* < 0.001). There was no difference for patients in the CTx cohort (36.6% vs. 29.3%, *p* > 0.05; **Figure 3A**). The representative images of patients in the IO cohort with different responses were illustrated in **Figure 3B**. Patients who were stratified into the good response group also showed better OS than those in the poor response group in the IO cohort (*p* = 0.001; **Figure 3C**). The survival of patients in the CTx cohort between the good and poor response groups was similar (*p* = 0.409).

### Performance of the prediction model in the external validation set

In the external validation set, 43 eligible patients were stratified using the prediction model into the good response group (n = 18) and the poor response group (n = 25). In the good response group, 14 patients achieved TRG0/1 (14/18, 77.8%), which was significantly higher than those in the poor response group (9/25, 36.0%, *p* = 0.012; **Figure 3D**). The performance indices of the prediction model between the IO cohort of the training set and the validation cohort were similar (**Table 2**).

**Table 2:** Performance indices of the prediction model to identify patients who achieved TRG0/1 after treatment of chemotherapy plus PD-1 antibody.

Indices	Training cohort (n = 120)	Validation cohort (n = 43)
AUC	0.728	0.639
Sensitivity (%)	67.3	60.9
Specificity (%)	72.3	80.0
Accuracy rate (%)	69.8	70.5
PPV (%)	67.3	77.8
NPV (%)	72.3	64.0

AUC, area under the receiver operating characteristic curve; NPV, negative predictive value; PPV, positive predictive value.

## Discussion

In this study, we established a clinical prediction model consisting of SMRA, NLR, and weight loss, which could effectively identify GC patients who would respond to chemotherapy plus PD-1 antibody. GC patients stratified as good responders by the prediction model showed a higher pathological response rate when treated with chemotherapy plus PD-1 antibody in both the training set and external validation set.

The selection of patients for treatment with PD-1 inhibitors is an important goal, as it prevents unnecessary immunotherapy-related complications and reduces medical costs. PD-L1 expression, microsatellite status, Epstein–Barr virus infection, and tumor mutational burden are currently being used to guide the application of PD-1 antibodies in GC patients (24, 25), while most of these biomarkers represent tumor characteristics. The role of patients’ phenotypes, which are also closely correlated with immune activity, remains under investigation in GC (26). Our study focused on patients’ body composition parameters and clinical factors that are easily accessible in clinical practice. TRG, the pathological indicator of treatment efficacy, was used as the efficiency outcome in the present study. Patients treated with chemotherapy alone during the same period were also included in the training set as a reference, which helped to assess the specificity of our clinical model.

SMRA is the body composition feature associated with pathological response in GC patients receiving chemotherapy plus PD-1 antibody. Associations between body composition parameters and clinical outcomes of immunotherapy have also been found in melanoma and lung cancer patients treated with PD-1 antibody with or without CTLA-4 antibody (27, 28). In a recent retrospective study, low SMI was identified as an independent risk factor for poor tumor regression in patients with advanced GC receiving chemotherapy plus PD-1 antibody; however, the role of SMRA was not analyzed (29). Our results extend the association of body composition parameters with immunotherapy in GC.

Integrating clinical factors with body composition parameters as a multivariate prediction model for PD-1 antibody-based therapy has not been performed in GC patients. Our data show that NLR and weight loss are both associated with pathological response in the IO cohort. NLR has been reported to be closely associated with the efficacy of PD-1 antibodies (30, 31). Proper energy and nutrition balance are essential for a healthy immune system and are commonly disrupted in gastrointestinal (GI) cancer patients due to cancer-related gastrointestinal symptoms (32). Skeletal muscle wasting, involuntary weight loss, and systemic inflammation are all features of cancer-associated cachexia (33). We hypothesized that the combination of these three parameters could provide more comprehensive information reflecting patients’ immune phenotypes. Indeed, our clinical model demonstrates a better stratification based on the combined adverse phenotypes, as is also verified in the external validation cohort. According to our results, GC patients who are stratified as good responders should be treated with chemotherapy plus PD-1 antibody. For those who are stratified as poor responders, the TRG0/1 rate between chemotherapy plus PD-1 antibody (27.7%) and chemotherapy alone seemed to be similar (32.9%), and other biomarkers should be assessed to predict treatment success. The present prediction model, based on immune phenotypes, has potential as a valuable tool to guide clinical decision making in the initiation of immunotherapy in patients with gastric cancer. This should be tested in a randomized controlled trial.

Low SMRA or myosteatosis is characterized by pathological fat accumulation in skeletal muscle and is related to cancer-induced systemic inflammation (34). Elevated inflammatory factors associated with myosteatosis can significantly impair the host’s antitumor immune response (35). For example, tumor-derived IL-6 induces muscle steatosis and dysmetabolism in pancreatic cancer (36). Increased IL-6 elevates serum glucocorticoid levels by suppressing hepatic ketogenesis, which inhibits intratumoral infiltration and proliferation of CD8^+^ T cells and results in immunotherapy resistance (37). Furthermore, TNF-α can compromise the functions of tumor-infiltrating CD8^+^ lymphocytes and induce PD-L1 expression on melanoma cells, promoting cancer immune escape (38). Conversely, myokines released by skeletal muscle cells, such as interleukin-15, participate in modulating the tumor immune microenvironment by promoting activities of natural killer cells and T cells (39, 40). Therefore, myosteatosis may not only be the result of systemic inflammation but may also impair the modulating effect of skeletal muscle on the tumor immune microenvironment, contributing to resistance to PD-1 antibody-based therapy.

Currently, multiple prediction models have been investigated for gastric cancer immunotherapy, including multi-omics analysis; however, the additive value of body composition parameter-based multivariate models has not been tested (41–43). This study provides a simple and efficient tool for clinicians to quickly obtain crucial information, allowing patients to receive timely treatment without waiting for complex, expensive, and time-consuming molecular tests. Despite the pressing need for highly accurate prediction models in precision oncology, body composition parameters, as highlighted by our findings, could be integrated into the multi-omics research to enhance treatment strategies.

There are some potential limitations of this study. The chemotherapy regimens are variable due to the retrospective nature; however, all these regimens are recommended by guidelines, which may reflect the real-world context. For either triplet or doublet regimens combined with PD-1 inhibitors, patients stratified using the prediction model into the good response group achieved a higher rate of TRG0/1. The cTNM stage was different between the training set and the validation set, but most of the patients had an advanced-stage disease, and the efficacy of the clinical model was verified in the validation set. The purpose of including patients treated with chemotherapy alone was to analyze the specificity of the clinical model to PD-1 antibody-based therapy rather than to compare the outcomes between the two cohorts. Moving forward, we plan to conduct a prospective trial to further validate the model’s predictive value, as well as to explore the underlying molecular mechanisms.

## Conclusion

A multivariate prediction model consisting of baseline SMRA, NLR, and weight loss was established and externally validated. The model could be used as an additional clinical tool to select GC patients who can benefit from chemotherapy plus PD-1 antibody.

## Data Availability

The original contributions presented in the study are included in the article/supplementary material. Further inquiries can be directed to the corresponding authors.

## Glossary

AGC: advanced gastric cancer
AUC: area under the receiver operating characteristic curve
BMI: body mass index
CT: computed tomography
CPS: combined positive score
GC: gastric cancer
irAEs: immune-related adverse events
L3: third lumbar vertebral
MSI-H: microsatellite instability high
NLR: neutrophil-to-lymphocyte ratio
OS: overall survival
PD-1: programmed cell death protein 1
ROC: receiver operating characteristic
SATI: subcutaneous adipose tissue index
SATRA: subcutaneous adipose tissue radiation attenuation
SMI: skeletal muscle index
SMRA: skeletal muscle radiation attenuation
TRG: tumor regression grade
VATI: visceral adipose tissue index
VATRA: visceral adipose tissue radiation attenuation
