# Supplementary material for: Clinical and body composition parameters as predictors of response to chemotherapy plus PD-1 inhibitor in gastric cancer
Source: Front Immunol. 2025 Oct 7;16:1685592. doi: 10.3389/fimmu.2025.1685592 (PMC12537701; doi:10.3389/fimmu.2025.1685592)
Supplement: Supplementary file 1 [file DataSheet1.pdf]

Title: Clinical and Body Composition Parameters as Predictors of Response to Chemotherapy plus PD-1 Inhibitor in Gastric Cancer

Figure S1 ROC analysis of the prediction model compared with other three parameters in the IO cohort

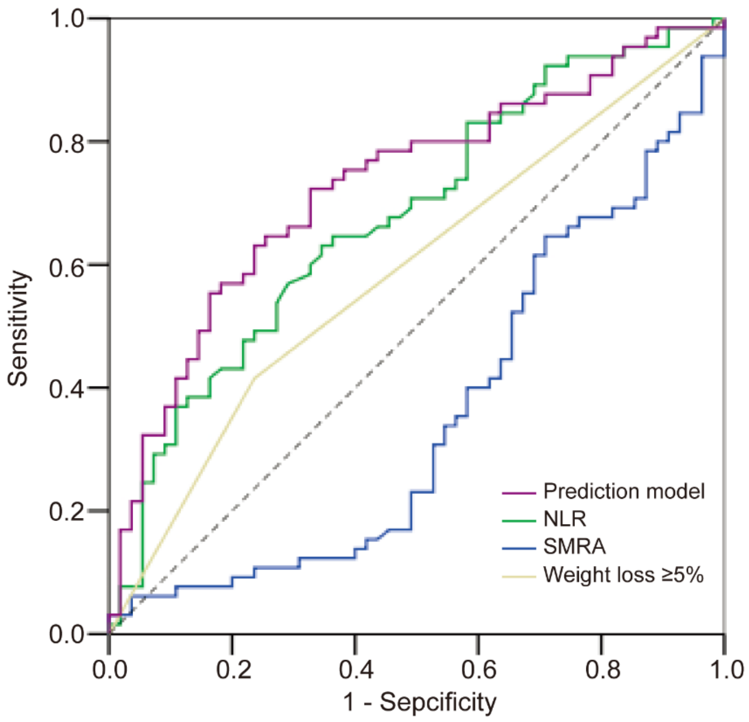

Table S1. Analysis of factors associated with TRG0/1 using univariate and multivariate logistic regression models

Parameters	IO cohort						CTx cohort					
	Univariate		*P* value	Multivariate		*P* value	Univariate		*P* value	Multivariate		*P* value
	OR	95% CI		OR	95% CI		OR	95% CI		OR	95% CI	
Female sex	0.923	0.422-2.020	0.842				2.290	0.859-6.107	0.098			
Age	0.990	0.959-1.022	0.541				0.958	0.918-1.000	0.052			
SMI	0.976	0.936-1.019	0.267				1.003	0.950-1.060	0.905			
VATI	0.997	0.982-1.013	0.743				1.009	0.987-1.032	0.418			
SATI	0.983	0.964-1.003	0.096				1.029	1.002-1.057	0.034	1.033	1.004-1.063	0.025
SMRA	0.950	0.908-0.994	0.026	0.947	0.902-0.994	0.027	0.990	0.930-1.054	0.756			
VATRA	1.009	0.981-1.037	0.533				0.985	0.938-1.034	0.539			
SATRA	1.008	0.988-1.029	0.421				0.967	0.933-1.002	0.063			
BMI	0.912	0.809-1.207	0.129				1.133	0.972-1.321	0.111			
Weight loss≥5%	2.296	1.038-5.087	0.040	2.116	0.903-4.959	0.085†	1.235	0.469-3.251	0.669			
ECOG 1	1.184	0.561-2.500	0.657				0.774	0307-1.953	0.588			
cTNM	2.027	0.970-4.236	0.060				1.905	0.690-5.260	0.214			
Metastasis	1.889	0.910-3.922	0.088				3.892	1.034-14.654	0.045	4.573	1.154-18.130	0.031
Prealbumin	1.000	0.993-1.006	0.908				1.004	0.994-1.014	0.412			
NLR	1.541	1.163-2.042	0.003	1.488	1.111-1.992	0.008	0.977	0.783-1.220	0.838			

BMI: body mass index; CTx: chemotherapy; CI: confidential interval; IO: immunotherapy; NLR: neutrophil to lymphocyte ratio; OR: odds ratio; SATI: subcutaneous adipose tissue index; SATRA: subcutaneous adipose tissue radiation attenuation; SMI: skeletal muscle index; SMRA: skeletal muscle radiation attenuation; TRG: tumor regression grade; VATI: visceral adipose tissue index; VATRA: visceral adipose tissue radiation attenuation;

†Weight loss was retained in the equation by using Enter method of multivariate analysis.

Table S2 Comparison of clinical parameters between the IO cohort and CTx cohort

Clinical parameters	IO cohort (range)	CTx cohort (range)	*P* value
Prealbumin (mg/L)	208.0 (21.0, 304.0)	207.5 (89.0, 354.0)	0.335
NLR	2.65 (0.65, 9.59)	2.38 (0.71, 13.91)	0.241
SMI (cm^2/m^2)	43.87 (28.79, 70.94)	40.47 (23.64, 68.17)	0.164
Male	47.85 (32.64, 70.94)	44.52 (23.64, 68.17)	0.304
Female	35.84 (28.79, 54.27)	37.27 (28.70, 55.42)	0.382
VATI (cm^2/m^2)	34.59 (0.0, 111.99)	27.22 (0.01, 110.03)	0.044
Male	38.78 (0.0, 111.99)	26.96 (0.15, 110.03)	0.044
Female	23.30 (2.17, 73.63)	28.44 (1.09, 78.14)	0.654
SATI (cm^2/m^2)	35.97 (0.2, 134.82)	37.54 (0.15, 111.65)	0.468
Male	33.58 (0.02, 69.84)	25.98 (0.15, 99.60)	0.272
Female	43.31 (20.24, 134.82)	46.98 (15.15, 111.65)	0.236
SMRA (HU)	47.59 (26.92, 72.55)	47.96 (28.50, 63.73)	0.942
Male	49.02 (30.08, 72.55)	49.03 (34.76, 63.73)	0.636
Female	45.68 (26.92, 70.09)	45.11 (28.50, 62.40)	0.981
VATRA (HU)	-90.26 (-125.0, -52.68)	-88.51 (-109.80, -55.50)	0.380
Male	-90.91 (-125.0, -52.68)	-86.73 (-109.80, -55.50)	0.298
Female	-88.97 (-114.50, -69.10)	-90.24 (-99.70, -69.83)	0.991
SATRA (HU)	-94.78 (-137.40, -32.75)	-94.54 (-121.70, -40.08)	0.961
Male	-93.86 (-128.80, -32.75)	-90.48 (-121.70, -40.08)	0.390
Female	-97.53 (-137.40, -71.83)	-98.73 (-113.80, -72.39)	0.968

CTx: chemotherapy; IO: immunotherapy; NLR: neutrophil to lymphocyte ratio; SATI: subcutaneous adipose tissue index; SATRA: subcutaneous adipose tissue radiation attenuation; SMI: skeletal muscle index; SMRA: skeletal muscle radiation attenuation; VATI: visceral adipose tissue index; VATRA: visceral adipose tissue radiation attenuation;

Table S3 Correlations among clinical parameters in the IO cohort

Parameters	SMI	VATI	SATI	SMRA	VATRA	SATRA	Weight	BMI	Weight loss	Prealbumin	NLR
SMI	1	.455(**)	.188(*)	.162	-.232(*)	-.099	.694(**)	.707(**)	-.093	.362(**)	-.093
VATI	.455(**)	1	.550(**)	-.239(**)	-.696(**)	-.495(**)	.652(**)	.767(**)	-.124	.319(**)	-.020
SATI	.188(*)	.550(**)	1	-.218(*)	-.576(**)	-.658(**)	.334(**)	.664(**)	-.254(**)	.179	-.089
SMRA	.162	-.239(**)	-.218(*)	1	-.143	-.152	-.070	-.125	.010	.116	-.090
VATRA	-.232(*)	-.696(**)	-.576(**)	-.143	1	.881(**)	-.425(**)	-.550(**)	.217(*)	-.300(**)	.053
SATRA	-.099	-.495(**)	-.658(**)	-.152	.881(**)	1	-.261(**)	-.432(**)	.208(*)	-.168	-.011
Weight	.694(**)	.652(**)	.334(**)	-.070	-.425(**)	-.261(**)	1	.859(**)	-.053	.304(**)	-.050
BMI	.707(**)	.767(**)	.664(**)	-.125	-.550(**)	-.432(**)	.859(**)	1	-.182(*)	.356(**)	-.086
Weight loss	-.093	-.124	-.254(**)	.010	.217(*)	.208(*)	-.053	-.182(*)	1	-.209(*)	.121
Prealbumin	.362(**)	.319(**)	.179	.116	-.300(**)	-.168	.304(**)	.356(**)	-.209(*)	1	-.325(**)
NLR	-.093	-.020	-.089	-.090	.053	-.011	-.050	-.086	.121	-.325(**)	1

** Correlation is significant at the 0.01 level (2-tailed).

* Correlation is significant at the 0.05 level (2-tailed).

BMI: body mass index; IO: immunotherapy; NLR: neutrophil to lymphocyte ratio; SATI: subcutaneous adipose tissue index; SATRA: subcutaneous adipose tissue radiation attenuation; SMI: skeletal muscle index; SMRA: skeletal muscle radiation attenuation; VATI: visceral adipose tissue index; VATRA: visceral adipose tissue radiation attenuation;
